# Contemporary insights into elamipretide’s mitochondrial mechanism of action and therapeutic effects

**DOI:** 10.1016/j.biopha.2025.118056

**Published:** 2025-04-27

**Authors:** Hani N. Sabbah, Nathan N. Alder, Genevieve C. Sparagna, James E. Bruce, Brian L. Stauffer, Luke H. Chao, Robert D.S. Pitceathly, Christoph Maack, David J. Marcinek

**Affiliations:** a Department of Medicine, Division of Cardiovascular Medicine, Henry Ford Hospital, Henry Ford Health, Detroit, MI, USA; b Department of Molecular and Cell Biology, University of Connecticut, Storrs, CT, USA; c Division of Cardiology, Department of Medicine, University of Colorado Anschutz Medical Campus, Aurora, CO, USA; d Department of Genome Sciences, University of Washington, Seattle, WA, USA; e Division of Cardiology, Department of Medicine, Denver Health and Hospital Authority, Denver, CO, USA; f Department of Molecular Biology, Massachusetts General Hospital, Boston, MA, USA; g Department of Genetics, Harvard Medical School, Boston, MA, USA; h Department of Neuromuscular Diseases, University College London Queen Square Institute of Neurology, London, UK; i NHS Highly Specialised Service for Rare Mitochondrial Disorders, Queen Square Centre for Neuromuscular Diseases, The National Hospital for Neurology and Neurosurgery, London, UK; j Comprehensive Heart Failure Center, University Clinic Würzburg, Würzburg, Germany; k Medical Clinic 1, University Clinic Würzburg, Würzburg, Germany; l Departments of Radiology and Laboratory Medicine and Pathology, University of Washington, Seattle, WA, USA

**Keywords:** Elamipretide, Cardiolipin, Mitochondria, Mechanism, MOA, Barth syndrome

## Abstract

Mitochondria are cellular hubs integral for metabolism, signaling, and survival. Mitochondrial dysfunction is centrally involved in the aging process and an expansive array of disease states. Elamipretide is a novel mitochondria-targeting peptide that is under investigation for treating several disorders related to mitochondrial dysfunction. This review summarizes recent data that expand our understanding of the mechanism of action (MOA) of elamipretide. Elamipretide is a potential first-in-class therapeutic that targets the inner mitochondrial membrane. Despite initial descriptions of elamipretide’s MOA involving reactive oxygen species scavenging, the last ten years have provided a significant expansion of how this peptide influences mitochondrial bioenergetics. The cardiolipin binding properties of elamipretide have been corroborated by different investigative teams with new findings about the consequences of elamipretide-cardiolipin interactions. In particular, new studies have shown elamipretide-mediated modulation of mitochondrial membrane electrostatic potentials and assembly of cardiolipin-dependent proteins that are centrally involved in mitochondrial physiology. These effects contribute to elamipretide’s ability to improve mitochondrial function, structure, and bioenergetics. In animal studies, elamipretide-mediated amelioration of organ dysfunction has been observed in models of cardiac and skeletal muscle myopathies as well as ocular pathologies. A number of clinical trials with elamipretide have been recently completed, and a summary of the results focusing on Barth syndrome, primary mitochondrial myopathy, and age-related macular degeneration, is also provided herein. Elamipretide continues to show promise as a potential therapy for mitochondrial disorders. New basic science advances have improved understanding of elamipretide’s MOA, enabling a better understanding of the molecular consequences of elamipretide-cardiolipin interactions.

## 1. Historical development of elamipretide

Mitochondria are organelles comprising intracellular networks that serve as a nexus between cellular stress, bioenergetics, and signaling [1]. They are composed of two membranes, inner and outer, with the inner mitochondrial membrane (IMM) forming invaginations called cristae where the mitochondrial electron transport system (ETS) and the oxidative phosphorylation system produce adenosine triphosphate (ATP) [2]. Changes in mitochondrial structure and function are a hall-mark of aging as well as myriad pathologies associated with chronic diseases [3,4]. However, given the central role of mitochondria in diverse cellular processes and the complex nature of mitochondrial structure and function, treatments targeting mitochondrial abnormalities remain a challenge.

Elamipretide (previously referred to as SS-31, MTP-131, or Bendavia) is a member of a class of small molecules that were discovered serendipitously when developing novel opioid receptor agonist peptides [5]. Elamipretide is similar to other small peptides with alternating cationic-aromatic motifs [6] that are cell-permeable and localize to the IMM, with little to no opioid receptor binding properties.

**Fig. 1.**
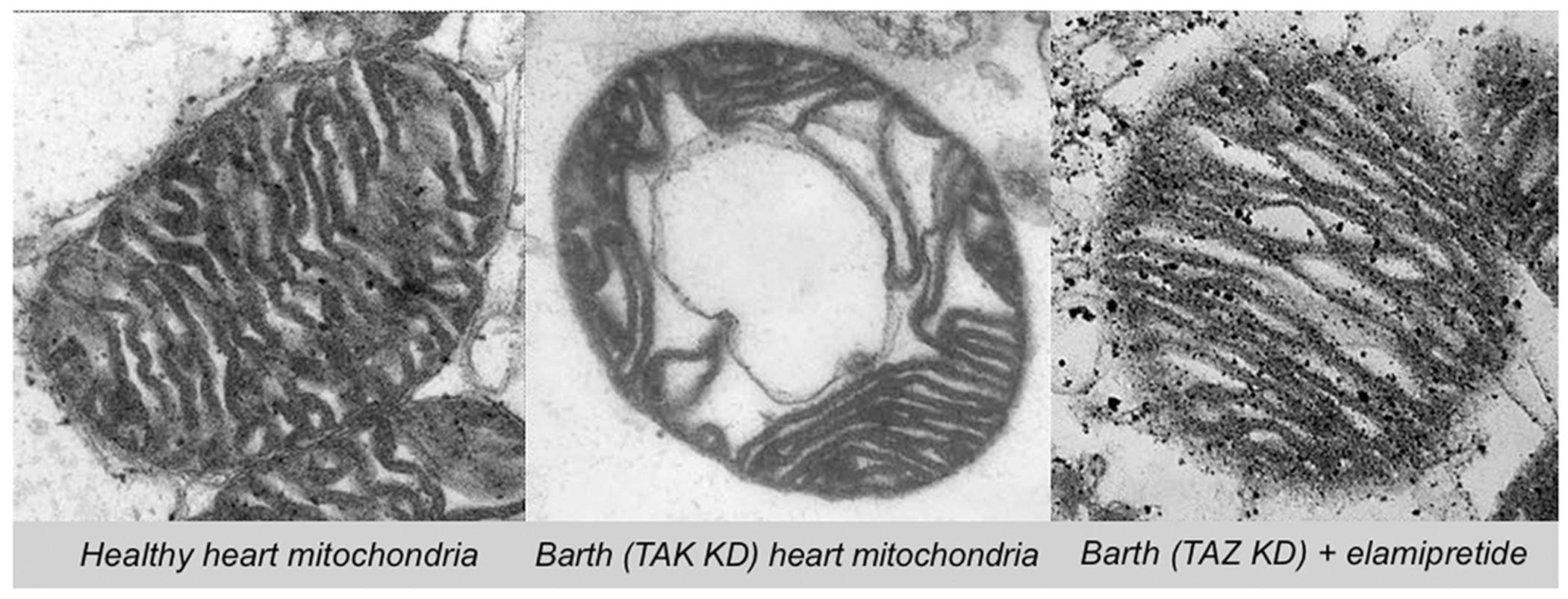
Representative transmission electron microscopy images of sections of cardiac mitochondria isolated from healthy (wildtype), diseased (*TAFAZZIN* knockdown), and elamipretide-treated disease mice [13].

Among this novel class of mitochondria-targeting peptides, elamipretide emerged as one of the most promising. In the two decades since its discovery, the use of elamipretide across disease models and understanding of its mechanism of action (MOA) have advanced substantially. Explored in over 150 peer-reviewed publications to date, elamipretide has been shown to localize to the IMM where cardiolipin (CL), a unique phospholipid involved in membrane and protein structure and function, is located. The robust mitochondrial protection is well-documented across preclinical models whereby mitochondrial structure and function are improved (summarized in Table 1 and Fig. 1). This review discusses recent discoveries related to the molecular mechanisms underlying elamipretide’s effect on mitochondria and how these effects may translate into clinical efficacy in the treatment of mitochondria-related diseases.

## 2. Elamipretide-cardiolipin interactions

The widely observed mitochondrial protection elicited by elamipretide has frequently been attributed to reducing reactive oxygen species (ROS) production and attenuating oxidative stress [16–18]. Although this property was originally postulated to be mediated by ‘scavenging’ ROS, subsequent studies have demonstrated a reduction of ROS emission by the ETS with no discernible radical scavenging properties [10,16]. This suggests that the MOA of elamipretide could involve improvement of mitochondrial function upstream of ROS production. Elamipretide also helps to sustain the IMM potential, which is crucial for ensuring cristae structure and sustaining bioenergetics [5,16].

Over the last ten years, it has become increasingly clear that elamipretide targets the IMM through an interaction with cardiolipin [19,20]. In recent years, there has been considerable progress in our understanding of elamipretide’s MOA. More insights have been provided related to elamipretide’s cardiolipin binding. Furthermore, there is a renewed appreciation for the consequences of the elamipretide-cardiolipin interaction, namely improved IMM protein assembly and function.

**Fig. 2.**
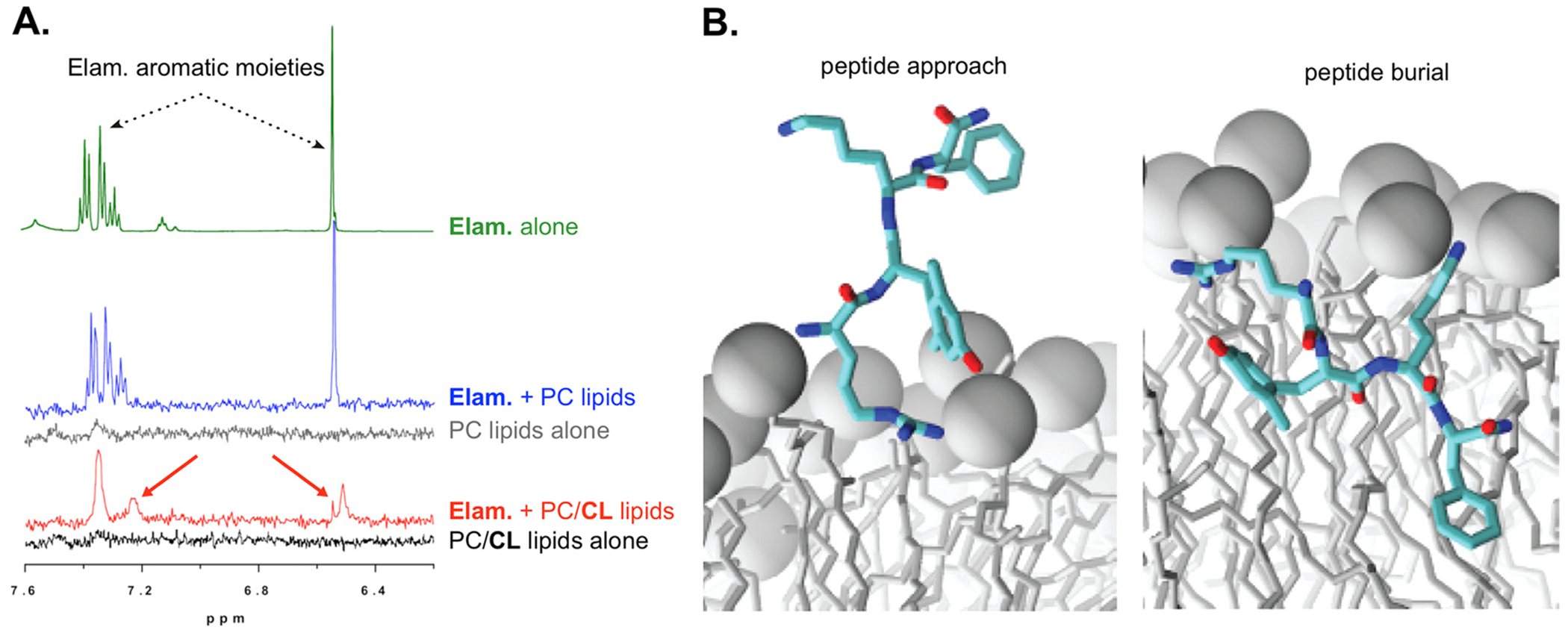
NMR-detected interaction of amino acids of elamipretide with (A) cardiolipin [20] and (B) elamipretide interactions with a CL-containing lipid bilayer evaluated by molecular dynamics simulations [21]. Abbreviations: CL = cardiolipin.

CL is an anionic phospholipid that plays a key role in mitochondrial bioenergetics including energy metabolism, apoptosis, signaling, membrane stability and dynamics, and fission/fusion dynamics [21–23]. Elamipretide targets the IMM where it binds selectively to CL (Fig. 2A) [20], with the association normalizing key CL properties that are modified during oxidative stress [17]. However, recent investigations are continuing to further elucidate the nature of the interaction between elamipretide and mitochondrial membranes.

Nuclear magnetic resonance (NMR) studies revealed that elamipretide binds to the IMM, IMM-mimetic model lipid vesicles, and isolated mitochondria, and that this binding is dependent on the presence of CL [10,21]. Further, this binding and the subsequent effects of elamipretide on membrane properties are dependent on CL acyl chains. Recent studies have focused on the modulating effects of elamipretide on CL-containing mitochondrial membrane surface charge [21,24,25]. Mitchell et al. used all-atom molecular dynamics simulations to analyze the interaction of elamipretide with lipid bilayers and observed that there are two states of interactions of elamipretide with the membrane: a “peptide approach” state in which positively-charged groups of the peptide become anchored to the negatively charged phosphates of lipid headgroups, and a “peptide burial” state in which the aromatic groups of the peptide partition into the nonpolar core of the membrane (Fig. 2B) [21]. Analyses of the elamipretide interactions with mitochondrial membranes suggest that the interaction is related more to surface charge than to the identity of the lipid headgroup. Critically, the peptide-bilayer interaction results in a “controlled down-tuning” of the negative charge density of the CL-rich bilayers, by likely two nonexclusive mechanisms: (i) through nonspecific addition of positive counterion charges to the headgroup region, and (ii) by specific chemical interactions between the basic peptide groups and lipid phosphates that partially neutralize negative charge density of the membrane [21].

This interaction between elamipretide and CL-containing lipid bilayers results in a unique MOA. The binding of elamipretide to CL results in a modulation of protein interactions with the IMM. This was first demonstrated for cytochrome c (CytC), wherein elamipretide reduced oxidative modifications of CL acyl chains by forming a complex with CL and inhibiting CytC peroxidase activity [20]. The mechanistic basis of this was further explored identifying how elamipretide partitions into the membrane interface, the chemically heterogeneous region containing lipid headgroups, water, and ions that separates bulk solvent from the nonpolar membrane core. The magnitude of the membrane surface charge, which originates primarily from negatively charged lipid headgroups, was found to directly modulate the lipid affinity and binding density of elamipretide [21,24]. Indeed, electrostatic interactions between the basic peptide and anionic lipid groups could constitute a key marker for the efficacy of this class of compounds [24]. Elamipretide produces a reduction in surface potential and dipole potential, while the transmembrane potential is not affected [21,25]. Anionic head groups play a key role in the creation of a strong electric field that increases the local concentration of elamipretide, thereby enhancing binding through hydrophobic contacts. Thus, the binding of elamipretide to CL entails a combination of electrostatic and hydrophobic interactions with a resultant reduction in negative surface charge and partial disordering of chemical interactions in the lipid headgroup region [21,24,25].

Elamipretide binding to CL affects membrane physical properties that are important across mitochondrial proteins involved in cristae formation, supercomplex association, and membrane transport [10,21,24,25]. These effects include changes in lipid bilayer properties, such as an increase in the self-association of CL, a decrease in the lateral diffusivity of lipids, and enhancements of lateral packing without a disruption of lamellar bilayers [21,24]. These effects potentially increase membrane curvature and membrane surface area, which could promote more efficient mitochondrial function [21]. The down-regulation of surface potential may also alter the physical properties of CL-containing bilayers, thereby reducing stresses on the mitochondria.

**Fig. 3.**
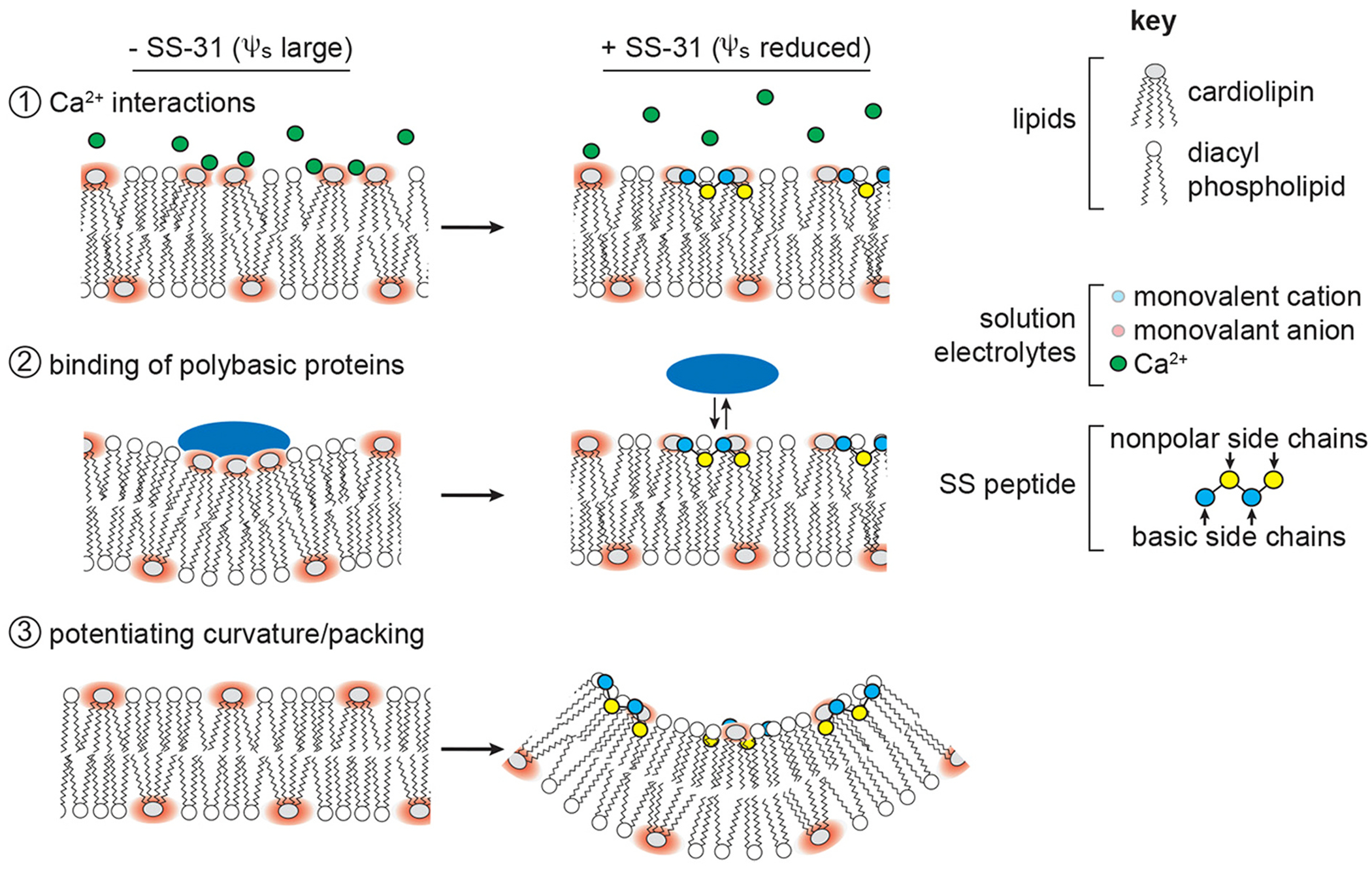
Proposed mechanisms by which the down-regulation of the surface potential on mitochondrial membranes alter lipid bilayer properties. (1) Alteration of the distribution of divalent cations, such as Ca^2+^, which interact strongly with CL. (2) Decrease in the mitotoxic interaction of basic proteins with CL-rich mitochondrial membranes through alteration of lipid bilayer properties, such as demixing and sequestration of CL, resulting in adverse effects on membrane integrity and lipid-protein interactions as well as lipid peroxidation. (3) Alterations in the physical properties of CL-containing bilayers [21]. Abbreviations: CL = cardiolipin; SS-31 = elamipretide.

Based on their results, Mitchell and colleagues proposed three working models of how the down-regulation of the surface potential of anionic mitochondrial membranes could explain the therapeutic efficacy observed with elamipretide (Fig. 3) [21]. First, reducing the surface potential could alter the distribution of divalent cations, such as Ca^2 +^, which interact strongly with CL, altering bilayer properties. Second, decreasing the magnitude of the surface potential could reduce the mitotoxic interaction of basic proteins such as CytC, which binds CL-rich membranes, causing lateral phase separation and peroxidation of unsaturated lipids. Finally, down-regulation of the surface potential could alter the physical properties of CL-containing bilayers through facilitating lipid packing and promoting local curvature [21].

## 3. Novel insights for elamipretide-inner mitochondrial membrane protein interactions

Chemical cross-linking mass spectrometry techniques have been developed to enable the identification of protein interactions from living cells and isolated functional organelles [2,26,27]. For example, chemical cross-linking with mass spectrometry identified elamipretide-mitochondrial protein interactions [2].

Mitochondria incubated with biotin-tagged elamipretide (b-ELAM) revealed 12 cross-linked proteins, all of which directly interact with CL. The interacting proteins can broadly be grouped into two classes: those that are involved in ATP production and those that are involved in 2-oxoglutarate metabolism. These results are consistent with the ability of elamipretide to improve mitochondrial bioenergetics. For example, elamipretide interacts with the complex IV (CIV) subunit NDUA4. Although originally considered to be a complex I subunit, NDUA4 was later identified as a CIV subunit [28] observed in CIV structures [29]. In addition, NDUA4 association with CIV inhibits the ability of the complex to form a dimer, where the monomer is the form that can incorporate into the supercomplex. Moreover, recent *in situ* cryo-electron microscopy studies with intact mitochondria consistently identified NDUA4 as an integral subunit in CIV in all four types of observed supercomplex assemblies [30]. Thus, the interaction of elamipretide with NDUA4 may be important for CIV incorporation in the supercomplex by inhibiting dimer formation [2]. b-ELAM treatment increased maximum uncoupled and CIV respiration, and decreased H_2_O_2_ production in mitochondria isolated from old mouse hearts [2].

**Fig. 4.**
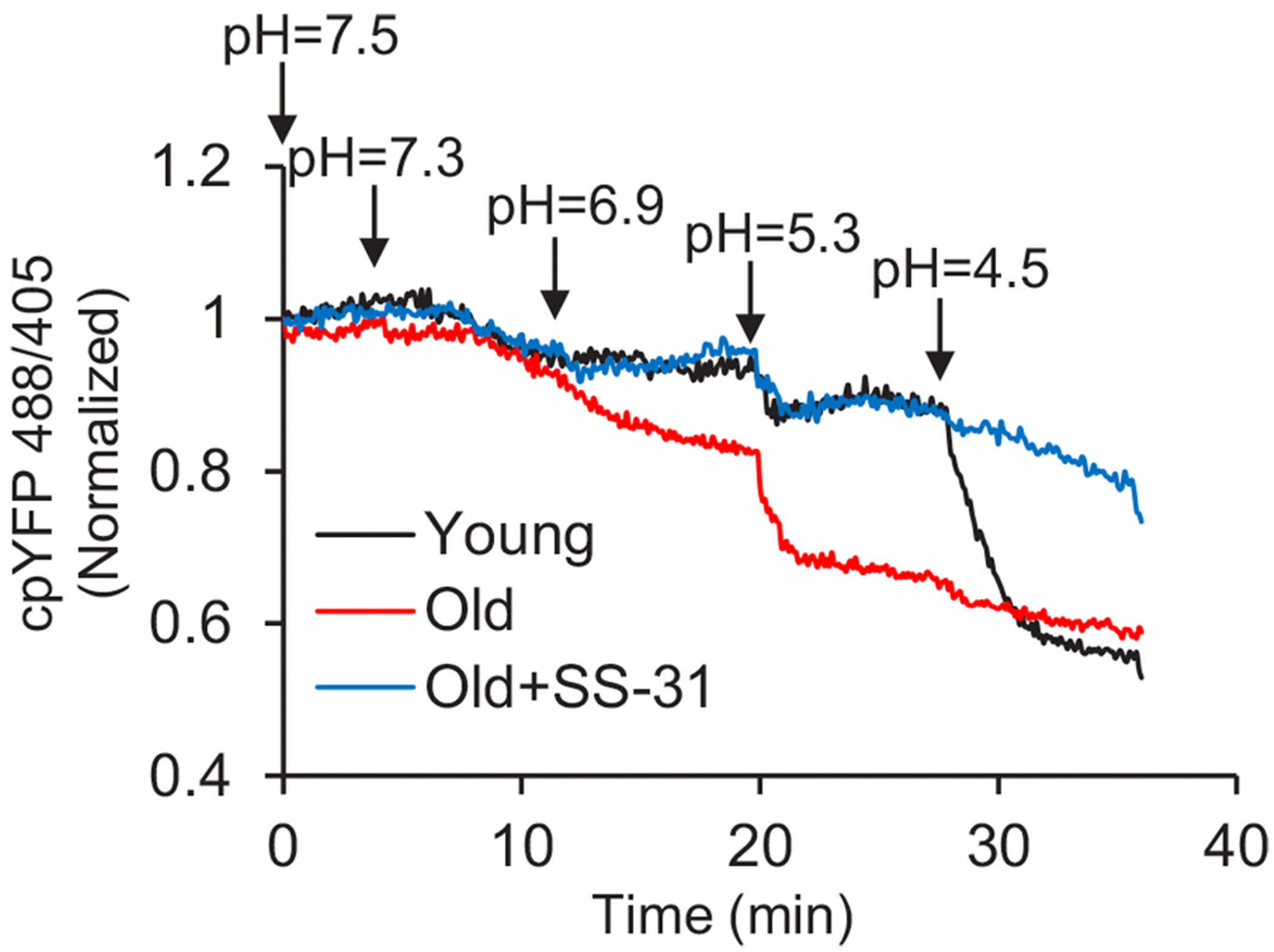
Permeabilized cardiomyocytes exposed to lower external pH demonstrated that permeability of old mitochondria was greater than that of young mitochondria, but elamipretide restored mitochondrial pH stress resistance in permeabilized aged cardiomyocyte mitochondria and reduced proton leakage [33]. Abbreviations: SS-31 = elamipretide.

Elamipretide also directly interacts with the adenosine diphosphate (ADP)/ATP translocase (ANT) 1. ANT1 is a transmembrane protein located in the IMM and is a carrier protein involved in the import of ADP from the cytosol. It also mediates the export of ATP from the mitochondrial matrix by cycling between the cytoplasmic-open state (c-state) and the matrix-open state (m-state). b-ELAM cross-links to the matrix face of ANT1 via two lysine residues [2]. Docking models suggest that b-ELAM binding to ANT1 in the m-state [2] is compatible with either the m-state or an alternate conformation as described by Bround et al. [31] and Caudal et al. [32]. The ANT1-elamipretide interaction has been independently verified via affinity purification experiments with Western blot, showing that elamipretide restores mitochondrial pH stress resistance in permeabilized aged cardiomyocyte mitochondria by reducing ANT1-mediated proton leakage (Fig. 4) [33]. Additional studies in aged skeletal and cardiac muscles revealed that elamipretide increased ADP sensitivity by increasing uptake of ADP through ANT, an effect that was associated with improved muscle force and cardiac pump function [34]. Thus, the interaction between elamipretide and ANT1 is associated with increases in ADP sensitivity, ADP transport by ANT1, and reduction of ANT1-derived proton leak [33,34]. Overall, these data suggest that the identified proteins that interact with elamipretide are functional components in oxidative phosphorylation, which is consistent with the ability of elamipretide to improve mitochondrial bioenergetics.

The improved bioenergetics extends to *in vivo* ATP production and resistance to cell stress by the mitochondria. Elamipretide rapidly improved mitochondrial function with a rapid reversal of age-related declines in *in vivo* resting and maximal mitochondrial ATP production, coupling of oxidative phosphorylation, and cellular energy state, as reported by the ratio of phosphocreatine over ATP [35]. In the same model, elamipretide also reduced age-related redox stress, restored resting redox homeostasis, and improved skeletal muscle function [36]. In the study noted above on ANT1 in aged cardiomyocytes, the reduced proton leak through the ANT1 in the presence of elamipretide also stabilized mitochondrial membrane potential and reversed the increased mitochondrial permeability transition pore opening, and restored mitochondrial function via interaction with ANT1 and stabilization of the ATP synthasome in rodent cardiomyocytes [33].

Improved mitochondrial energetics have also been shown *in vivo* in humans. In a randomized placebo-controlled trial in healthy older adults, a single dose of elamipretide increased mitochondrial energetic capacity relative to placebo as evidenced by an increase in the maximal rate of ATP production (ATPmax) in skeletal muscle [37].

### 3.1. Electron transport system supercomplex interactions (Table 2)

In addition to direct binding with ANT1, elamipretide improves the activity of ETS supercomplexes. In normal mitochondria, the respiratory complexes I-IV are assembled into supercomplexes that also interact with fatty acid oxidation enzymes through the trifunctional protein [38]. In mitochondrial diseases (eg, Barth syndrome [BTHS]), these supercomplexes are destabilized and reduced in number, resulting in decreased efficiency of this metabolic pathway. The binding of elamipretide promotes the assembly of respiratory supercomplexes and enhances electron transfer, which is associated with improvements in mitochondrial function, remodeling of mitochondria, and promotion of tissue regeneration during aging [11]. Elamipretide also stabilizes the ATP synthasome, a protein supercomplex that includes ANT, ATP synthase, creatine kinase, and an inorganic phosphate carrier [33].

Following cardiac ischemia-reperfusion in rats, elamipretide ameliorated cardiac ischemia-reperfusion induced reductions in supercomplex coupling and mitigated fragmentation of cristae networks. These effects were accompanied by improved structural and functional characteristics, including increased mitochondrial density, increased cristae connectivity, enhanced cristae networking within and between mitochondria, and improved inner membrane integrity [10].

The beneficial effect of elamipretide on ETS complexes has also been demonstrated in several disease states. BTHS is a genetic disease that is caused by loss-of-function mutations in the *TAFAZZIN* gene that remodels mitochondrial CL. Loss of tafazzin results in a dysregulation of CL biosynthesis and maturation, resulting in an increase in monolysocardiolipin (MLCL). BTHS is characterized by childhood onset of cardiomyopathy, skeletal myopathy, neutropenia, and growth retardation [38].

**Fig. 5.**
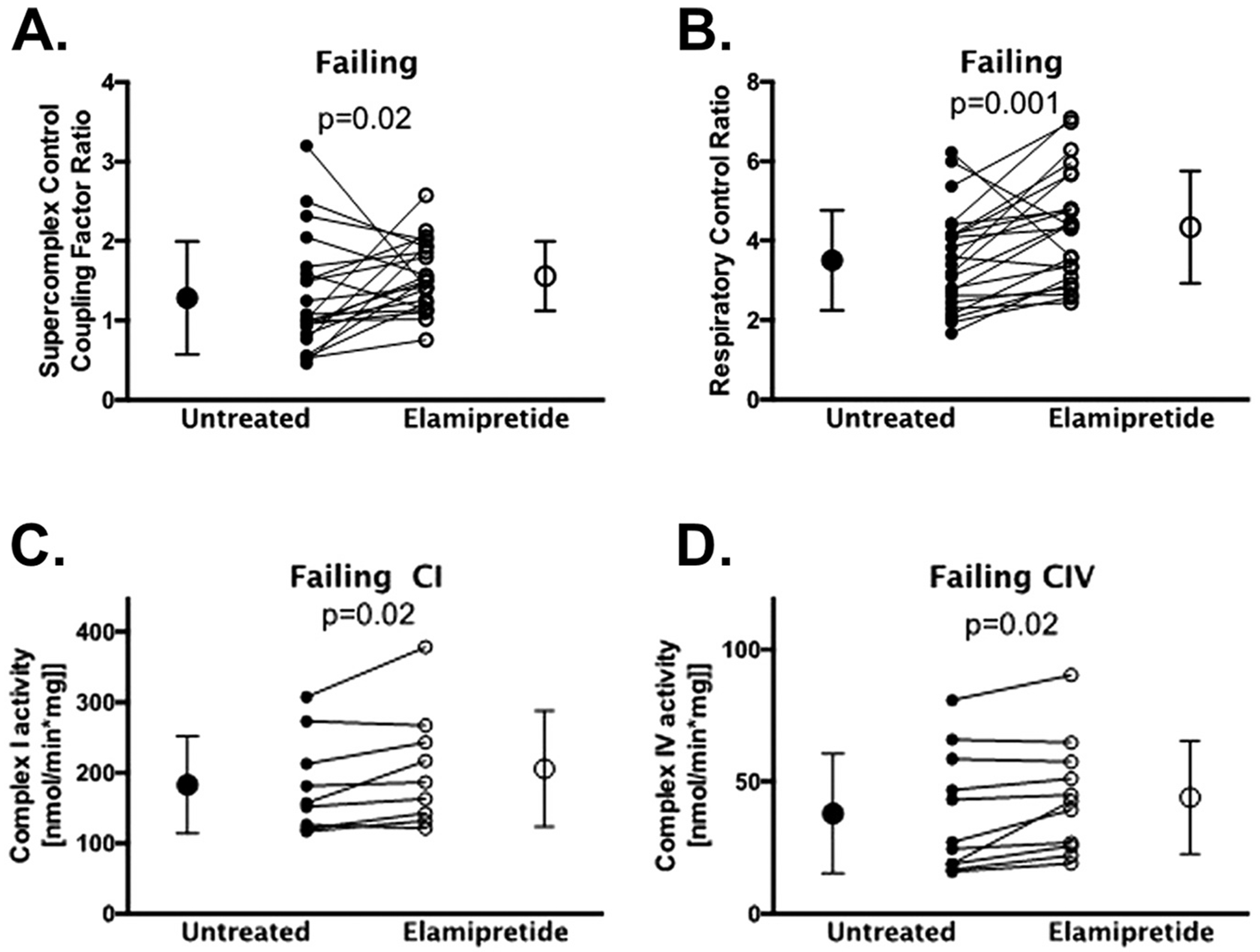
Mitochondrial function is impaired in explanted failing pediatric and human hearts. In explanted tissue from patients with heart failure, elamipretide was associated with improvements in supercomplex coupling control factor (Panel A), respiratory control ratio (Panel B), CI activity (Panel C), and CIV activity (Panel D) [12]. Abbreviations: CI = complex I; CIV = complex IV.

In *TAFAZZIN* knockdown mice, elamipretide improved mitochondrial respiratory capacity and promoted supercomplex organization [7]. *Ex vivo* studies using explanted ventricular tissue from failing human hearts showed that mitochondrial function was impaired using in-gel activity assays to assess ETS components within the mitochondrial supercomplex and in parallel by using high resolution respirometry on intact mitochondria [12]. Treatment with elamipretide improved the coupling of supercomplex-associated enzyme complexes I, III, and IV as well as improvements in oxygen flux across a diverse mix of human heart failure etiologies (Fig. 5) [12]. Improvements in mitochondrial and supercomplex function occurred with the acute treatment protocol without remodeling of CL which is consistent with the changes in membrane properties discussed above. However, long-term treatment with elamipretide in dogs and humans with heart failure resulted in normalized heart failure-associated abnormalities in CL synthesis and CL remodeling, and reversed the dysregulation of the fusion and fission proteins, peroxisome proliferator-activated receptor gamma coactivator alpha (PGC-1α), and mitofilin [40].

### 3.2. Prohibitin interaction

In aged mice, elamipretide was recently shown to interact with prohibitin 2, resulting in an improved mitochondrial turnover and improvements in neural damage [39]. The effects were mediated by an inhibition of the cGAS-STING pathway and M1 microglial polarization. Notably, the protective effect of elamipretide was abolished by knockdown of prohibitin 2 [39].

## 4. Consequences of targeting cardiolipin-enriched domains in disorders of mitochondrial dysfunction

Given the effects of elamipretide on mitochondrial structure, dynamics, and function, it is not surprising that the drug elicits favorable physiologic effects in multiple preclinical models of mitochondrial dysfunction. Because elamipretide selectively binds to cardiolipin, it is effective only in diseases of mitochondrial dysfunction where cardiolipin abnormalities exist. Several studies have demonstrated that elamipretide reverses cardiac, skeletal, and kidney dysfunction in various preclinical disease models that also manifest mitochondrial dysfunction.

### 4.1. Models of cardiac dysfunction

Cardiomyopathy and heart failure are characterized by destabilization of ETS supercomplex formation, resulting in changes in mitochondrial dynamics and ultrastructure, decreased ATP synthesis, overproduction of ROS, and dissociation of fatty acid oxidation enzymes [40,9,8,14]. In fibroblasts from patients with dilated cardiomyopathy with ataxia syndrome (DCMA), a mitochondrial cardiomyopathy, elamipretide reversed mitochondrial fragmentation and excessive ROS production and increased levels of the long form of the mitochondrial cristae-remodeling protein optic atrophy 1 (Opa1) [8,9]. The latter is associated with CL and is important in maintenance of mitochondrial membrane ultrastructure [8,9].

In old mice with left ventricular (LV) diastolic dysfunction, eight weeks of treatment with elamipretide normalized LV diastolic function, increased the ratio of early-to-late diastolic mitral annulus velocities (Ea/Aa), improved exercise tolerance with regression of cardiac hypertrophy, and normalized mitochondrial proton leak and ROS formation [41]. Elamipretide also reversed systolic dysfunction in aged mice with recovery of age-related declines in global LV longitudinal strain, LV fractional shortening, and LV ejection fraction compared to baseline, along with functional capacities restored to levels similar to that of young mice [34].

In a porcine model of hypercholesterolemic renovascular hypertension that was associated with CL loss, myocardial injury, and LV diastolic dysfunction, treatment with elamipretide was associated with improved LV relaxation and amelioration of LV cardiac hypertrophy without affecting blood pressure or systolic LV function [15]. Similarly, long-term treatment with elamipretide improved LV systolic and mitochondrial function in dogs with microembolization-induced advanced heart failure [14]. This included improvements in LV ejection fraction (EF) and prevention of progressive LV dilatation. The magnitude of effect was similar to that observed with long-term therapy with angiotensin-converting enzyme inhibitors and beta blockers [14]. Elamipretide also reduced N-terminal pro-brain natriuretic peptide (NT-proBNP) and proinflammatory cytokines, including tumor necrosis factor-α and c-reactive protein, and restored mitochondrial function (eg, increased ATP synthesis and reduced ROS formation) [14]. Overall, these data suggest that by improving overall mitochondrial function, elamipretide elicits clinically relevant improvements of LV function and remodeling.

The nicotinamide nucleotide transhydrogenase (NNT) communicates between the nicotinamide adenine dinucleotide plus hydrogen (NADH) and nicotinamide adenine dinucleotide phosphate (NADPH) pools in mitochondria and normally regenerates NADPH from NADH. Experimental chronic cardiac pressure overload excessively increases ATP demand at the myofilaments and thereby oxidizes NADH towards ATP production at the respiratory chain. This reverses the NNT reaction to regenerate NADPH from NADH, but at the cost of the NADPH-coupled antioxidative capacity. Therefore, chronic pressure overload increases mitochondrial ROS emission, which leads to necrosis, apoptosis, contractile dysfunction, and death [42]. Chronic *in-vivo* treatment with elamipretide efficiently prevented this pressure overload-induced necrosis and apoptosis, and also prevented premature death of the animals [42]. Furthermore, in hypertrophic cardiomyopathy (HCM), variants in genes encoding sarcomeric proteins commonly increase the Ca^2+^ affinity of myofilaments. This also causes an energetic mismatch with NADH oxidation and via reverse-mode NNT, also of NADPH, and the ensuing increase in ROS causes arrhythmias by providing triggers through spontaneous Ca^2+^ release events from the sarcoplasmic reticulum, and a substrate for arrhythmias by slowing ventricular conduction [43]. Elamipretide prevents ROS-induced slowing of ventricular conduction and thereby, may prevent arrhythmias in HCM [43].

### 4.2. Models of skeletal muscle dysfunction

In preclinical models evaluating age-related declines in skeletal muscle mitochondrial function, treatment with elamipretide improved skeletal muscle function [34,36]. This included improvements in skeletal muscle energetics and fatigue resistance as well as increased exercise endurance [36]. In elderly humans with reductions in mitochondrial function, a single dose of elamipretide elevated ATPmax in skeletal muscle, and post-hoc analyses indicated increased muscle fatigue resistance [37]. In canines with chronic heart failure, long-term elamipretide treatment restored skeletal muscle fiber-type composition to a more normal type, likely leading to improved exercise tolerance [44]. Furthermore, *ex vivo* exposure of skeletal muscle mitochondria to elamipretide improved or even normalized all measures of mitochondrial function in a dose-dependent manner [44].

### 4.3. Models of age-related macular degeneration

Declines in mitochondrial function are also implicated in changes of retinal pigment epithelial (RPE) cells that are characteristic of age-related macular degeneration (AMD) [45]. In a mouse model of visual aging, elamipretide had a protective effect on the RPE and improved visual function [46–48]. In RPE cell cultures from human donors with AMD, elamipretide was associated with improvements in RPE cell viability and mitochondrial function [46,47]. In mice with age-related visual impairments, treatment with elamipretide resulted in the mitigation of visual decline and the normalization of visual acuity and contrast sensitivity within two months [48].

## 5. Clinical advances with elamipretide

The positive effects of elamipretide on mitochondrial structure, dynamics, and function are likely the basis for the observed drug’s favorable effect on multiple mitochondria-related disorders in a variety of clinical trials. Elamipretide has been evaluated in several mitochondria-related diseases, including primary mitochondrial myopathy, BTHS, and AMD. While overall clinical results have been mixed, the results underscore the difficulties associated with assessing mitochondria-targeted therapies.

### 5.1. Barth syndrome experiences

In a Phase 2/3 randomized double-blind, cross-over clinical trial (TAZPOWER) in 12 patients with BTHS, 12 weeks of treatment with elamipretide did not meet the primary endpoints of improvements in 6Minute Walk Test (6MWT) and the Barth Syndrome Symptom Assessment (BTHS-SA) scale [49]. However, in the open-label phase, elamipretide was associated with significant improvements in 6MWT and BTHS-SA as well as several secondary endpoints, such as knee extensor strength, patient global impression of symptoms, and some cardiac parameters. The lack of effect at 12 weeks may reflect the need for longer-term therapy for skeletal and cardiac muscle remodeling to occur. This is supported by the results of a natural history comparison assessing the efficacy of elamipretide in eight patients from TAZPOWER who completed at least 72 weeks of the open-label phase versus untreated natural history control patients with BTHS [50]. The results indicated that elamipretide was associated with significant improvements in the 6MWT versus controls with least squares (LS) mean differences of 79.7m at Week 64 (*P*=0.0004) and 91.0m at Week 76 (*P*=0.0005) [50]. Statistically significant improvements were also observed for secondary functional endpoints for muscle strength, as assessed by handheld dynamometry and the time to complete the Five Times Sit-to-Stand Test. These results suggest that long-term therapy with elamipretide may attenuate the natural decline in heart function and improve functional capacity in this rare and difficult-to-treat disease.

### 5.2 Age-related macular degeneration

The efficacy of elamipretide in the treatment of AMD was evaluated in a Phase 2 randomized placebo-controlled, double-blind trial (ReCLAIM-2) in patients aged ≥55 years with AMD with non-central geographic atrophy (GA) [51]. Elamipretide treatment did not meet the primary endpoint (mean change in low-luminance best-corrected visual acuity [LL-BCVA] and mean change in the square root converted GA area) but was associated with a 43% reduction in the mean progression from baseline in the macular percentage of total ellipsoid zone (EZ) attenuation and a 47% reduction in the mean progression of macular percentage of partial EZ attenuation. The effect on EZ attenuation may be clinically relevant because progression to total EZ attenuation/photoreceptor loss precedes pathological changes associated with AMD progression, such as geographic atrophy, leading to irreversible vision loss. A post-hoc analysis of ReCLAIM-2 quantitatively assessed the effect of elamipretide on EZ integrity and its relationship with visual function [52]. Elamipretide was associated with a significantly greater reduction in the progression of partial EZ attenuation (15% elamipretide vs 34% placebo; *P*=0.01) and in total EZ attenuation (26% elamipretide vs 48% placebo; *P*=0.04). In addition, more patients experienced a ≥2-line gain in LL-BCVA (14.6% elamipretide vs 2.1% placebo; *P*=0.04). Further, LL-BCVA change at the end of the study correlated with the change in the macular percentage of total EZ attenuation (r=−0.35; *P* < 0.0001).

### 5.3. Primary mitochondrial myopathy

MMPOWER-3 was a randomized, double-blind, placebo-controlled trial in 218 patients with primary mitochondrial myopathy, which is a group of genetic disorders that predominantly affect skeletal muscle [53]. Eligible patients were randomized to receive either elamipretide 40mg/day or daily placebo for 24 weeks with the primary endpoints being change from baseline on the distance walked on the 6MWT and the Total Fatigue Score on the Primary Mitochondrial Myopathy Symptom Assessment (PMMSA). The results indicated that elamipretide was generally well tolerated with mild to moderate injection site reactions being the most commonly reported adverse events. The drug did not meet the primary endpoints (6MWT and PMMSA Total Fatigue Score) in the intent-to-treat population, however, analysis by genetic subgroups revealed that patients with nuclear DNA mutations (but not those with mitochondrial DNA mutations) achieved a significantly greater improvement from baseline in the 6MWT for those receiving elamipretide compared to the placebo group. These findings warrant further investigation as they highlight the importance of considering genetic subtypes when evaluating mitochondrial directed therapies.

## 6. Discussion

Mitochondria are membrane-bound organelles that have a primary role in cellular metabolism and energy production. They are also important mediators of cellular growth and differentiation, cell signaling, and cell apoptosis [54]. The clinical importance of mitochondria is reflected in the wide spectrum of diseases that are associated with abnormalities in mitochondrial structure and function. Notably, mitochondrial diseases are clinically heterogenous and can affect various organs and tissues including those that are highly dependent on aerobic metabolism [3] even though the underlying causes of mitochondrial diseases extend beyond a reduction in ATP production [55]. This heterogeneity of roles of mitochondria in the cell and the complex pathophysiology of mitochondrial diseases underscore the complexity of mitochondrial diseases and add to the challenge of managing these disorders. Thus, the conventional therapeutic strategy of designing drugs that target a single protein is unlikely to be effective in restoring mitochondrial function to mitigate disease pathology.

By localizing to the IMM and associating with CL, elamipretide improves assembly of ETS complexes into respiratory supercomplexes and as a result, respiratory efficiency [55,56], thereby improving bioenergetics, reducing ROS production, and improving mitochondrial morphology in dysfunctional mitochondria.

Recent studies show that elamipretide interacts with proteins that are associated with the IMM, interact with CL, and are involved in oxidative phosphorylation. Elamipretide has also been recently shown to modulate mitochondrial membrane surface electrostatic properties, which may underpin its modulation of electron carrier properties and its ability to interact with ANT and ETS supercomplexes. These changes of membrane physical properties can themselves elicit improvements in overall mitochondrial function that are independent of alterations in constituent protein structure and function, and therefore reinforces elaimpretide’s ability to target mitochondrial health at multiple levels within the organelle itself.

Preclinical models of disease also show that elamipretide improves mitochondrial structure and function, enhances mitochondrial bioenergetics, and is associated with improvements in physiologic functions, such as cardiac and skeletal muscle performance, as well as improvements in age-related retinal dysfunction. Clinical trials also support elamipretide’s efficacy in mitochondrial-related diseases (eg, BTHS, mitochondrial myopathy, and AMD), although the results have been mixed, which may be due to a variety of factors including the difficulty in identifying appropriate endpoints, small patient populations, and inadequate treatment duration. Nevertheless, it is clear that mitochondria-targeted agents have a bright future in the treatment of a diverse range of mitochondrial diseases. Studies that expand our understanding of the physiological underpinnings of mitochondrial diseases and the molecular and cellular effects of mitochondria-targeted agents, such as elamipretide, can help build greater knowledge for the search and discovery of effective drugs that address the unmet need we face in the clinical management of mitochondrial diseases.

## Figures and Tables

**Fig. 6.**
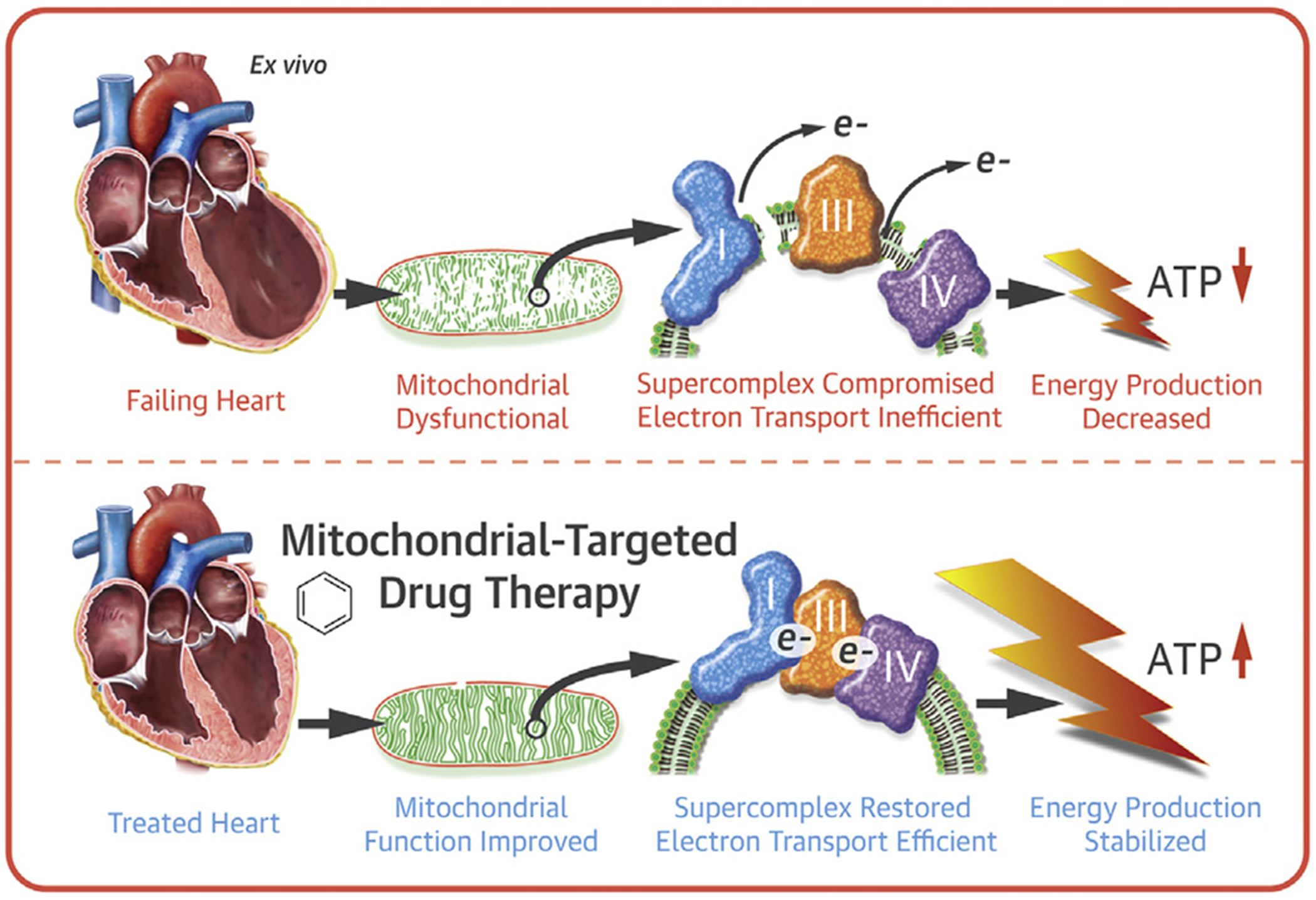
Model of the effect of elamipretide affects supercomplex assembly and ETS efficiency [12]. Abbreviations: ATP = adenosine triphosphate.

**Table 1:** Pre-clinical Preservation of Mitochondrial Ultrastructure and Function.

Model	Improvement with elamipretide treatment	Reference
**Improved Mitochondria Ultrastructure**		
Tafazzin-knockdown mice (Barth syndrome model)	Mitochondrial ultrastructure	Russo, et al. 2024 [7]
Mitochondrial cardiomyopathy patient cells	Mitochondrial cristae and mitochondrial content	Rohani, et al. Can J Cardiol. 2020 [8]; Machiraju, et al. Front Cardio Med. 2019 [9]
Rat cardiac ischemia-reperfusion	Cristae density and networking	Allen, et al. Nature Comm Biol. 2020 [10]
Aged mouse: heart and retinal mitochondria	Mitochondrial cristae density	Szeto and Liu. Archiv Biochem Biol. 2018 [11]
Diabetic mouse retinopathy	Mitochondrial ultrastructure	Szeto and Birk. 2014 [5]
**Improved Mitochondrial Function**		
Explanted pediatric and adult heart mitochondria	Mitochondrial respiration supercomplexes	Chatfield, et al. 2019 [12]
Explanted human heart mitochondria (HCM)	Mitochondrial respiration	Nollet, et al. Euro Heart Journal. 2023
Tafazzin-knockdown mice (Barth syndrome model)	Cardiac mitochondrial respiration and supercomplexes	Russo, et al. 2022 [13]
Canine model of heart failure	Mitochondrial respiration, ATP generation, membrane potential	Sabbah, et al. Circ Heart Fail. 2016 [14]
Pig model of heart failure with preserved EF	Mitochondrial respiration, lowered ROS emission	Eirin, et al. JAHA. 2016 [15]
Rat cardiac ischemia-reperfusion	Myocardial complex l, ll, and lV-dependent respiration	Allen, et al. 2020 [10]

HCM = hypertrophic cardiomyopathy; EF = ejection fraction.

**Table 2:** Mitochondrial proteins shown to be affected by elamipretide-cardiolipin interaction.

Inner Membrane Protein Influenced by Elamipretide	Cardiolipin Dependency for Protein Assembly/Function?	References
Respiratory supercomplexes and electron transport system subunits	Yes	Allen 2020 [10]; Szeto 2018 [11]; Zhang 2020 [33]; Pharaoh 2023 [34]; Chatfield 2019 [12]; Russo 2022 [7]; Chavez 2020 [2]
Adenine nucleotide translocator (ANT)	Yes	Pharaoh 2023 [34]; Zhang 2020 [33]
Prohibitin	Yes	Ji 2024 [39]

## Data Availability

All data in this review was collected from the literature.

## Glossary

6MWT: 6Minute Walk Test
ADP: adenosine diphosphate
AMD: age-related macular degeneration
ANT1: ADP/ATP translocase
ATP: adenosine triphosphate
ATPmax: maximal rate of ATP production
b-ELAM: biotin-tagged elamipretide
BTHS: Barth syndrome
BTHS-SA: Barth Syndrome Symptom Assessment
CIV: complex IV
CL: cardiolipin
c-state: cytoplasmic-open state
CytC: cytochrome c
DCMA: dilated cardiomyopathy with ataxia syndrome
Ea/Aa: ratio of early-to-late diastolic mitral annulus velocities
EF: ejection fraction
ES: electron transport system
EZ: ellipsoid zone
GA: geographic atrophy
HCM: hypertrophic cardiomyopathy
IMM: inner mitochondrial membrane
LL-BCVA: low-luminance best-corrected visual acuity
LS: least squares
LV: left ventricle/ventricular
MOA: mechanism of action
MLCL: monolysocardiolipin
m-state: matrix-open state
NADH: nicotinamide adenine dinucleotide plus hydrogen
NADPH: nicotinamide adenine dinucleotide phosphate
NMR: nuclear magnetic resonance
NNT: nicotinamide nucleotide transhydrogenase
NT-proBNP: N-terminal pro-brain natriuretic peptide
Opa1: optic atrophy 1
PGC-1α: peroxisome proliferator-activated receptor gamma coactivator alpha
PMMSA: Primary Mitochondrial Myopathy Symptom Assessment
ROS: reactive oxygen species
RPE: retinal pigment epithelial
SS-31: elamipretide
